# Akt Signaling in Macrophage Polarization, Survival, and Atherosclerosis

**DOI:** 10.3390/ijms20112703

**Published:** 2019-06-01

**Authors:** MacRae F. Linton, Javid J. Moslehi, Vladimir R. Babaev

**Affiliations:** 1 Atherosclerosis Research Unit, Division of Cardiovascular Medicine, Department of Medicine, Vanderbilt University School of Medicine, 2220 Pierce Avenue, Nashville, TN 37232-6300, USA; javid.moslehi@vumc.org; 2 Department of Pharmacology, Vanderbilt University School of Medicine, 2220 Pierce Avenue, Nashville, TN 37232-6300, USA

**Keywords:** atherosclerosis, macrophages, Akt signaling, polarization, apoptosis, survival

## Abstract

The PI3K/Akt pathway plays a crucial role in the survival, proliferation, and migration of macrophages, which may impact the development of atherosclerosis. Changes in Akt isoforms or modulation of the Akt activity levels in macrophages significantly affect their polarization phenotype and consequently atherosclerosis in mice. Moreover, the activity levels of Akt signaling determine the viability of monocytes/macrophages and their resistance to pro-apoptotic stimuli in atherosclerotic lesions. Therefore, elimination of pro-apoptotic factors as well as factors that antagonize or suppress Akt signaling in macrophages increases cell viability, protecting them from apoptosis, and this markedly accelerates atherosclerosis in mice. In contrast, inhibition of Akt signaling by the ablation of Rictor in myeloid cells, which disrupts mTORC2 assembly, significantly decreases the viability and proliferation of blood monocytes and macrophages with the suppression of atherosclerosis. In addition, monocytes and macrophages exhibit a threshold effect for Akt protein levels in their ability to survive. Ablation of two Akt isoforms, preserving only a single Akt isoform in myeloid cells, markedly compromises monocyte and macrophage viability, inducing monocytopenia and diminishing early atherosclerosis. These recent advances in our understanding of Akt signaling in macrophages in atherosclerosis may have significant relevance in the burgeoning field of cardio-oncology, where PI3K/Akt inhibitors being tested in cancer patients can have significant cardiovascular and metabolic ramifications.

## 1. Introduction

Atherosclerosis is a slowly progressive inflammatory disease and the underlying cause of heart attack and stroke [1,2]. Macrophage-derived foam cells are the major cell types in early atherosclerotic lesions and key features of unstable plaques. The performance and efficiency of the protective functions of macrophages in atherosclerotic lesions depends on their capability for activation [3] and their ability to survive in the toxic environment of atherosclerotic lesions [4]. Recent observations strongly suggest that PI3K/Akt signaling is a crucial modulator of both cell polarization and survival. Here, we discuss and summarize recent updates regarding the role of PI3K/Akt signaling in macrophage polarization and survival and in the pathogenesis of atherosclerosis.

## 2. PI3K Signaling and Akt Isoforms in Macrophages

PI3K signaling is downstream of multiple cell-surface receptors that regulate cell proliferation, survival, and death [5]. Activation of receptor tyrosine kinases and growth factors induce PI3K activity with the formation of heterodimers of class Ia lipid kinases, which are composed of catalytic and regulatory subunits. These heterodimers initiate the production of the lipid second messengers, phosphatidylinositol-3,4-biphosphate (PIP_2_) and phosphatidylinositol-3,4-triphosphate (PIP_3_). Importantly, PIP_3_ exists transiently and is normally rapidly metabolized by phosphatase and tensin homolog (PTEN), which ends PI3K signaling via the removal of the 3′-phosphate from PIP_3_. PTEN has been shown to act as a dual-specificity protein phosphatase that dephosphorylates lipid substrates like PIP_3_, in this case negatively regulating the PI3K signaling cascade [6]. Another negative regulator of PI3K/Akt signaling is SH2-containing inositol phosphatase (SHIP), which is responsible for the synthesis of PIP_2_. Activation of PI3K initiates the Akt phosphorylation at two key residues, T^308^ and S^473^ [7]. Phosphorylation of Akt on T^308^ is both necessary and enough to induce Akt signaling, whereas phosphorylation of S^473^ is required for maximal activation of the kinase [7]. Akt directly controls the mammalian target of rapamycin (mTOR) complex 1, which is a key sensor of nutrient signaling, and it regulates cellular metabolism, translation, and cytokine responses [8,9]. A recent logical modeling approach indicates that PI3K also controls mTOR complex 2 (mTORC2), but more than one regulator is necessary to explain the behavior of mTORC2 [10], which directly regulates AktS^473^ phosphorylation [11]. Together, mTORC1 and mTORC2 are major regulators of growth that control cell metabolism in response to nutrient-induced signals [9]. Importantly, pharmacological and genetic inhibition of mTOR increases the lifespan in multiple model organisms [12]. PI3K/Akt signaling has perhaps been best studied in tumorigenesis, where activating PI3K mutations or inactivating PTEN mutations represent frequent mutations in various cancer types. Currently, large numbers of PI3K/Akt inhibitors are being tested for the treatment of several cancer types. Perhaps less appreciated are the roles that the PI3K/Akt pathways play in macrophages and atherosclerosis.

There are three Akt isoforms in macrophages, Akt1, Akt2 and Akt3. They are products of different genes [13] and have similar structures displaying high (80%) homology and considerable differences between the isoforms in the last 130 amino acids [14]. Several studies have utilized a gene-targeting approach with knockouts of the individual Akt isoforms in mice to provide evidence for the isotype-specific functions of Akt. For example, Akt1 knockout mice exhibited augmented perinatal mortality and decreased in body weight [15,16]. Mice deficient for the *Akt2* gene had normal growth, but they acquired a diabetes-like syndrome with hyperglycemia and insulin resistance [16]. For comparison, loss of the Akt3 isoform in mice reduced brain weight with decreases of both cell size and cell numbers, but maintained relatively normal glucose homeostasis and body weight [17]. These reports demonstrate that each Akt isoform has differential or non-redundant physiological functions [5,7].

### Macrophage Phenotypes in Atherosclerosis

Macrophages can exhibit two clear functional phenotypes: Inflammatory or classically activated M1 and alternatively activated M2 macrophages [13,18,19,20]. M1 macrophages may be induced by treatment with interferon-gamma (IFNγ) or the toll-like receptor (TLR) 4 ligand, lipopolysaccharide (LPS). In contrast, alternatively activated M2 macrophages may be generated by treatment with interleukin (IL)-4 or IL-13 [19,21]. These M2 macrophages show an immunosuppressive phenotype with increased proliferation, substantial scavenging activity, and production of anti-inflammatory cytokines. Both phenotypes of macrophages can be reversibly shifted in different cytokine environments [22]. Actually, there are significantly more polarization statuses that can be defined according to the activation stimulus, and they are specific for different diseases and conditions [23]. As generally accepted, M1 macrophages have a crucial impact in plaque initiation, progression, and instability [24], whereas M2 macrophages are implicated in the resolution of inflammation and regression of atherosclerosis [25]. Therefore, priming macrophages to the M1 or M2 phenotype significantly affects their inflammatory abilities [19] and modulates the development of atherosclerosis [3,19,24].

In atherosclerotic lesions, M1 and M2 macrophages are derived primarily from different subsets of blood monocytes and local tissue-resident macrophages [3]. M1 macrophages are primarily derived from the inflammatory Ly6C^hi^ blood monocyte subset. Alternatively activated M2 macrophages typically originate from Ly6C^lo^ subsets, but also from Ly6C^hi^ subsets of monocytes [26]. Importantly, M1 macrophages produce MCP-1, IL-12, IL-23, and TNF, which are all crucial for recruitment and protection from alien organisms. In contrast, M2 macrophages express IL-10, arginase I, and chemokines, which play crucial roles in the resolution of inflammation, wound healing, and tissue remodeling [26]. These data demonstrate that macrophage phenotypes are complex and dynamic conditions with possible alteration during the different stages of atherosclerosis.

## 3. Impact of Akt Signaling on Macrophage Polarization

It is widely accepted that PI3K/Akt signaling mediating via mTORC1 regulates the effector responses of macrophages that affect innate immune responses [27] and has a direct effect on macrophage polarization [28]. A recent concept suggests that Akt-mTORC1 signaling in macrophages and dendritic cells modulates polarization, and the M1 pro-inflammatory phenotype is generated by a switch to high anaerobic glycolysis, fatty acid synthesis, and a truncated citric acid cycle compared to oxidative phosphorylation specific for M2 macrophages [29,30,31]. Early reports have indicated that PI3-kinase plays a negative role in the process of macrophage activation and have suggested that this enzyme might suppress the action of anti-inflammatory cytokines [32]. Alternatively, activation of the PI3K/Akt pathway may play a critical role in the restriction of pro-inflammatory responses in LPS-stimulated macrophages [33,34]. Here, we highlight more recent data mainly obtained with knockout mice that may clarify the role of Akt signaling in macrophage polarization and its impact on atherogenesis.

It is well known that PI3K/Akt activation significantly contributes to macrophage polarization with subsequent stimulation or suppression the immune response [5]. For example, deficiency of tuberous sclerosis 1 induces constitutive mTORC1 activation in macrophages, and this generates the inflammatory M1 phenotype resistant to M2 polarization stimuli [35]. In contrast, *Raptor* deficiency in macrophages, and respectively the disruption of mTORC1, reduces chemokine gene expression [36]. Similarly, mTORC1 deficiency in hematopoietic cells diminishes myelopoiesis and suppresses some innate immune responses in these cells [37]. Thus, Akt-mTORC1 signaling in macrophages leads to increased histone acetylation and the induction of a subset of genes supporting the M2 phenotype [38].

Arranz and co-authors [39] demonstrated that Akt isoforms play important roles in macrophage polarization: Deficiency of the Akt1 isoform induces M1 cells, while the loss of the Akt2 isoform generates the M2 phenotype. Indeed, deficiency of Akt1 promotes M1 activation, which enhances bacterial clearance [39,40]. These effects of Akt1 in macrophages are, at least partially, mediated via the induction of miR-155 and the suppression of C/EBPβ, a transcription factor of M2 differentiation and a critical regulator of the immunosuppressive environment [41]. In contrast, *Akt2*^−/−^ monocytes and macrophages display the M2 phenotype and express significantly lower levels of inflammatory genes and high levels of IL-10 in response to LPS [39,42]. In addition, *Akt2*^−/−^ blood monocytes exhibited suppressed migration in response to MCP-1 and dramatically reduced ability for IFNγ-mediated induction of the chemokine C-C motif receptor 2 [42]. Importantly, chemokine C-C motif receptor 2 is crucial for the recruitment of monocytes into atherosclerotic lesions [43]. Therefore, LDL receptor deficient (*Ldlr*^−/−^) mice transplanted with *Akt2*^−/−^ hematopoietic cells had less atherosclerotic lesions than the control *Ldlr*^−/−^ reconstituted with wild-type (WT) marrow [42]. Consistently, Akt2/*Ldlr* double deficient mice exhibited less atherosclerosis [44], and in a more recent study where *Ldlr*^−/−^ mice were transplanted with *Akt2*^−/−^ bone marrow, they had reduced levels of atherosclerosis [45]. Remarkably, Akt1 and Akt2 isoforms exhibited opposing functions in several other experiments including Rac/Pak signaling and cell migration [46,47], cell invasion assays, and the metastasis of tumor cancer [48,49]. These data indicate that the balance Akt1 and Akt2 isoforms controlling mTORC1 activity is important in PI3K/Akt-mediated polarization of macrophages.

Apart from Akt isoform-specific macrophage polarization, the level of PI3K/Akt activity is also critical in the process. For example, deficiency of *Ship*, a negative regulator of PI3K/Akt signaling, significantly enhances the M2 phenotype in peritoneal and alveolar macrophages [50]. Thus, enhanced PI3K/Akt signaling generates M2 macrophage differentiation. Indeed, more recent studies have shown that the loss of *Ship* in macrophages promotes the M2 phenotype and diminishes inflammatory cytokine production [51,52]. As a result, innate and adaptive immune responses in *Ship*^−/−^ cells were inhibited in these cells [53]. Similarly, deficiency of Pten, another negative regulator of PI3K, markedly increased Akt signaling and induced M2 macrophage markers [54,55,56]. In contrast, macrophages isolated from mice with a myeloid-specific Rictor deletion (M-*Rictor*^−/−^), which completely destroys mTORC2 assembly, have suppressed Akt signaling in blood monocytes, neutrophils, and peritoneal macrophages [57]. These M-*Rictor*^−/−^ macrophages were skewed to the M1 phenotype and expressed low levels of IL10 [58]. Thus, suppression of Akt activity in macrophages generates the M1 phenotype.

Similarly, M1 phenotype macrophages were found in *Ldlr*^−/−^ mice reconstituted with IκB kinase (IKK) alpha deficient hematopoietic cells [59]. IKK contains two catalytic subunits, IKKα and IKKβ, and a regulatory IKKγ subunit. Interestingly, IKKβ has higher affinity to the regulatory protein NF-κB and can form homodimers in an in vitro binding assay [60]. This is consistent with the fact that an *Ikkα* null mouse has a slightly milder phenotype and dies soon after birth compared to an *Ikk*β knockout mouse, which is embryonic lethal [61]. IKKβ initiates the well-defined classical NF-κB pathway, whereas IKKα has been implicated in the alternative pathway [60,62]. Loss of IKKα in macrophages significantly suppressed NF-κB signaling and inflammatory response in these cells [63]. In addition, IKKα is also required for B-cell maturation and the formation of secondary lymphoid organs [64]. Importantly, IKKα is associated with Rictor and regulates mTORC2 activity [65]. A recent report demonstrated that IKKα interacts with mTORC2 to regulate PI3K/Akt activity and positively promotes Akt phosphorylation at Ser^473^ [66]. Therefore, *Ikk*α^−/−^ macrophages suppressed mTORC2 signaling and reduced Akt S^473^ phosphorylation in mouse macrophages [59]. Thus, low levels of Akt signaling induce the inflammatory M1 phenotype, and high levels of Akt activity induce alternative M2 macrophages. These data also suggest the dual role of IKKα in inflammation. Activation of IKKα phosphorylates IκBα, and release NF-κB complexes to the nucleus, which initiates an inflammatory reaction. At the same time, IKKα phosphorylation induces Rictor-mediated activation of mTORC2 and the phosphorylation of Akt S^473^ with amplification of Akt signaling that, eventually through mTORC1, suppresses inflammation [59,66]. Since these two different roles for IKKα signaling differ in terms of the length of time for the onset of their effects, this second role for IKKα in amplifying Akt signaling remained hidden for a long time.

## 4. Macrophage Apoptosis and Atherosclerosis

Macrophage apoptosis significantly influences atherosclerosis formation at every stage of disease including early lesions, plaque progression, and plaque stability [67,68]. The current concept of apoptosis suggests that changes in environmental factors initiate perturbations in the endoplasmic reticulum to induce activation of ER-specific unfolded protein response (UPR) [69,70,71]. To overcome ER stress, the cells activate UPR, which controls several cellular pathways, including (a) reducing total protein synthesis, (b) upregulating ER chaperone protein expression, and (c) activating the ER-associated degradation arm of UPR. Together, these mechanisms decrease the ER burden by reducing its misfolded protein content [72,73,74]. Excessive and long-lasting ER stress activates different branches of UPR, eventually inducing apoptosis [75]. Therefore, changes in pathways that induce apoptosis in macrophages may significantly increase the cellularity of atherosclerotic lesions. For example, the loss of Bax, a pro-apoptotic factor, in hematopoietic cells significantly increased early atherosclerosis in *Ldlr*^−/−^ mice [76]. In contrast, deficiency of the apoptosis inhibitor of macrophages (AIM) increased macrophage apoptosis and suppressed early atherosclerosis in *Ldlr*^−/−^ mice [77]. These data support the notion that macrophage apoptosis regulates cellularity and decreases lesion growth in early atherosclerosis [78]. Therefore, the repression of apoptosis increases atherosclerotic lesions and enlarges the necrotic core [75]. Numerous studies have shown that decreased levels of apoptosis in macrophages significantly increased the size of early atherosclerotic lesions and were related with reduced plaque burden in more advanced lesions [72,79,80]. Together, these data suggest that prevention of UPR and ER stress may have a different impact than just blocking specific apoptosis signaling pathways.

## 5. PI3K/Akt Signaling and Cell Survival

The role of PI3K/Akt signaling in cell survival was extensively described in several reviews [5,13,81]. There are multiple mechanisms that suppress apoptosis through the direct phosphorylation of anti-apoptotic factors (Bad, Caspase) or via activation of the transcriptional genes (MDM2, IKK, Yap) supporting cell survival [81]. Activation of Akt signalling phosphorylates Bad at Ser 136 and promotes its binding to cytosolic 14-3-3 proteins, which prevents Bad from inhibiting the anti-apoptotic molecule BCL-XL [82]. We have shown previously that this mechanism is significantly compromised in mouse macrophages deficient in the prostaglandin E_2_ receptor, EP4 [83]. Akt signalling inhibits the Forkhead family of transcription factors FoxO, which suppresses the Bcl-2 family member Bim and inhibits apoptosis [84]. Moreover, Akt signaling modulates cell survival by controlling the BH3-only protein, murine double minute-2, and an E3 ubiquitin ligase that triggers p53 degradation [85]. Acting through glycogen synthase kinase, Akt may inhibit an anti-apoptotic member of the Bcl family, Mcl-1 [86]. A recent report has shown that *Akt2*^−/−^ mice with induced hepatic Akt1 deletion developed spontaneous hepatocellular carcinoma, which is associated with FoxO-dependent liver injury and inflammation [87]. In addition, Akt signalling suppresses activity of the pro-apoptotic factor Bax, keeping the permeability of mitochondrial membranes under control and, thereby, inhibiting apoptosis [88]. Finally, Akt signaling regulates the activity of GSK3 isoforms and caspase-9 [88]. Together, these results indicate that Akt signaling activity controls cell survival ability in multiple ways.

PI3K/Akt signaling regulates the resistance of macrophages to apoptotic stimuli and, therefore, is a crucial determinant of atherosclerotic plaque cellularity [5,81]. It is critical to understand the mechanisms of the survival pathways that protect macrophages from apoptosis. Akt signaling is constitutively active in human and mouse macrophages [89], and the inhibition of Akt signaling induces cell apoptosis [83,89]. The loss of a single isoform, including Akt1 or Akt2, in embryonic fibroblasts [20] and mouse macrophages [42], as well as Akt3 deficiency [90], has no impact on apoptosis. Direct comparison all four types of WT, *Akt1*^−/−^, *Akt2*^−/−^, and *Akt3*^−/−^ macrophages did not show any differences in responses to pro-apoptotic stimuli [91]. Remarkably, the loss of Akt1 in apoprotein E null macrophages increased apoptosis [92], whereas deficiency in the Akt1 or Akt2 isoform did not impact this process [42], suggesting that apoprotein E may interact with Akt to promote cell survival.

**Figure 1:**
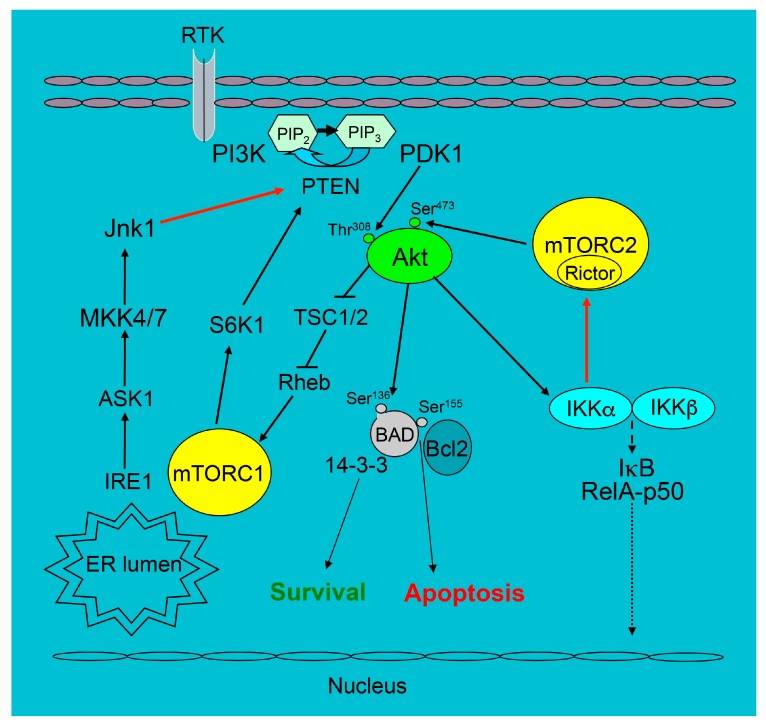
The survival signaling pathways related to the PI3K/Akt pathway in mouse macrophages. The black arrows show the direction of signaling, T-bars indicate the suppression of following signaling and dotted arrows are possible phosphorylation of IκB with release the complex into nucleus whereas the red arrows are the part of signaling we discussed in our review.

Changes in Akt signaling of macrophages modify cell survival and this directly affects atherosclerosis. For instance, c-Jun NH_2_-terminal kinase (JNK) is a direct mediator of UPR activity [93], and one of the JNK isoforms, JNK1, antagonizes Akt signaling, suppressing its activity [94]. Therefore, the loss of JNK1 in macrophages protects them from apoptosis, and *Ldlr*^−/−^ mice reconstituted with JNK1 null bone marrow showed increased early atherosclerosis [95]. In contrast, when Akt signaling is diminished, macrophage survival is suppressed. As example, IKKα is directly related to two major pro-survival pathways, NF-κB and PI3K/Akt. During an inflammatory response, NF-κB activates several pro-survival genes that antagonize apoptosis induced by TNFα, including the JNK cascade [96,97]. The PI3K/Akt pathway acting via IKKα triggers a part of anti-apoptotic gene expression of the NF–κB pathway [98,99,100]. When the role of IKKα in macrophage survival and atherosclerosis was investigated, it turned out that both deficiency of IKKα and pharmacologic inhibition of IKK suppress Akt phosphorylation in macrophages. Therefore, IKKα-deficient macrophages exhibited markedly diminished survival under conditions of ER stress, and this dramatically decreased blood monocyte and macrophage viability [59]. Consequently, *Ldlr*^−/−^ mice with IKKα^−/−^ hematopoietic cells had increased apoptotic macrophages in atherosclerotic lesions and reduced lesion size compared to *Ldlr*^−/−^ mice reconstituted with WT hematopoietic cells [59]. Although Telstam and co-workers [101] have reported that the knock-in of a non-achievable IKKα kinase (*Ikkα*^AA/AA^) in the bone marrow of ApoE^−/−^ mice had no effect on atherosclerosis, we would like to stress here that these *Ikkα*^AA/AA^ mice had no defects specific for the loss of the IKKα function in the original descriptions of the paper regarding these mice [102]. Thus, suppression of Akt signaling in IKKα-deficient macrophages reduces their survival and diminishes early atherosclerosis. A schematic illustration of some pro-survival signaling related to the PI3K/Akt pathway is shown in Figure 1.

It is important to note that *Ikkα* deficiency, besides suppressing Akt signaling, has the opportunity to enhance macrophage activation, because, according to Lawrence et al. [63], IKKα suppresses inflammation. This possibility restricted our understanding regarding the role of mTORC2 in macrophages. Consequently, in the next set of experiments, *Ldlr*^−/−^ mice were transplanted with *Rictor* knockout hematopoietic cells to examine whether the loss of mTORC2 impacts monocyte and macrophage viability and whether it affects atherosclerosis. We observed that the loss of Rictor disrupts mTORC2 assembly and downstream signaling, significantly inhibiting Akt activity, and this diminishes monocyte and macrophage survival, consequently decreasing early atherosclerosis. These results stress the significance of macrophage Akt signaling in atherogenesis.

## 6. Double Akt Isoform Knockout Mice Highlight Critical Pro-Survival Role of Akt1

Experiments with double Akt knockout mice clearly show the important and dose-dependent role of Akt signaling in the survival of mice. The loss of a single Akt isoform is generally well tolerated and does not affect the survival of mice with a single knockout. In contrast, the loss of two Akt isoforms generates detrimental effects in mice [103]. For example, double *Akt1/Akt3*-deficient mice do not survive with embryonic lethality (at 11–12 days) as this double knockout creates harsh developmental defects in the cardiovascular and nervous systems [104]. Remarkably, *Akt1*^−/−^/*Akt3*^+/−^ mice had many defects in the thymus, heart, and skin and died soon after birth, whereas *Akt1*^+/−^/*Akt3*^−/−^ mice survived normally, indicating that the Akt1 isoform is more critical than Akt3 for embryonic development and survival [104]. Similarly, *Akt1/Akt2* knockout mice had early lethality after birth with impaired skin and bone development and reduced adipogenesis [105]. In contrast, *Akt2/Akt3*-deficient mice survived both the embryonic and postnatal periods, but they later developed insulin intolerance and a reduction in body weight compared to wild-type mice [106]. Remarkably, the presence of a single allele of Akt1 (embryonic development of *Akt1*^+/-^*Akt2*^−/−^*Akt3*^−/−^) in mice appears to be sufficient for survival [106]. Together, these data support the idea that Akt1 is the critical pro-survival Akt isoform [89,92,107]. However, it remains unclear what the specific functions of the Akt1 isoform are that make it so important for mouse survival.

## 7. Loss of Two Akt Isoforms in Hematopoietic Cells Is Detrimental for Their Survival

The PI3K/Akt pathway plays crucial roles in the viability of hematopoietic cells and a number of reports indicate that Akt signaling promotes both the maturation and the survival of thymocytes [108,109] and peripheral B cells [110]. For these reasons, mice reconstituted with *Akt1/Akt2* knockout hematopoietic cells had developmental defects of thymocytes in terms of survival, proliferation, and differentiation [111,112]. Mao and coworkers concluded that Akt1, Akt2, and, to a lesser extent, Akt3 contribute to the development of thymocytes and the loss of Akt1/Akt2 inhibits their survival [112]. Similarly, double Akt1/2 knockout follicular B cells demonstrated a survival defect in a competition assay against wild-type B cells in vivo [110]. Interestingly, the loss of the AKT1 or AKT2 isoform had only a minimal effect on hematopoietic stem cell function, whereas AKT1/2 double-deficient cells exhibited long-term functional defects that were caused by decreased reactive oxygen species production and survival [113]. Together, these data demonstrate that the loss of two Akt isoforms in hematopoietic cells produces devastating effects on the viability of these cells.

## 8. Mice with a Single Akt Isoform in Hematopoietic Cells Exhibited Low Levels of White Blood Cells, B-Cells, and Monocytes, and Increased Apoptosis in Monocytes and Macrophages

Recently, Liu and co-authors [20] demonstrated that a small portion of Akt is required for usual cell activity and survival of mouse embryonic fibroblasts under normal conditions, whereas more Akt activity is necessary when cells are under different forms of stress. They also suggested that this threshold of Akt inhibition is inherent and variable in different types of cells. It remained unclear, however, whether a similar threshold of Akt inhibition pertained in macrophages. Given the crucial role of macrophage survival in the pathogenesis of atherosclerosis, defining the impact of reduced Akt expression by monocytes and macrophages on the development of atherosclerosis is a fundamentally important goal.

To further elucidate whether marked reduction of the Akt protein to a single isoform may compromise the viability of monocytes and macrophages, we generated mice expressing only the Akt1 (Akt1^only^) or the Akt3 (Akt3^only^) isoform in hematopoietic cells [91]. This novel approach allowed us to compare the impact of expression of the individual Akt1 and Akt3 isoforms on macrophage viability and atherosclerosis. 

Remarkably, both groups of mice reconstituted with Akt1^only^ or Akt3^only^ hematopoietic cells exhibited dramatic reductions in white blood cell counts and monocytopenia. In addition, Akt1^only^ or Akt3^only^ monocytes and macrophages of these mice had markedly increased sensitivity to apoptotic stimuli, and treatment with the JNK inhibitor, SP600125, significantly reversed viability in these macrophages [59]. As a result, male *Ldlr*^−/−^ mice reconstituted with Akt1^only^ or Akt3^only^ bone marrow cells had significantly smaller atherosclerotic lesions compared to mice with WT cells. Thus, the reduction of the Akt protein to a single isoform suppressed monocyte and macrophage viability, which significantly diminished early atherosclerosis, and the loss of two Akt isoforms markedly compromised cell survival, with higher levels of resistance to apoptosis in macrophages expressing Akt1 compared to cells with the Akt3 isoform [91].

Previously, we have shown that the loss of Akt1 in macrophages significantly suppressed *Il10* gene expression [42], and this suggested that the Akt1 isoform may play a role in the regulation of the gene. Indeed, Akt1^only^ macrophages produced significantly higher (three-fold) levels of *Il10* gene expression than WT and Akt3^only^ cells [91]. When exogenous IL-10 was added to cells alone, it had no impact on apoptosis, however, when IL-10 was combined with the lipotoxic pro-apoptotic factor palmitic acid [114], every cell type, including WT, Akt1^only^, and Akt3^only^ macrophages, benefited significantly with dramatic suppression (two-fold) of apoptosis [91]. Thus, IL-10 protects cells from apoptosis, and high levels of *Il10* gene expression in Akt1^only^ macrophages likely contribute to their survival advantage over Akt3^only^ cells.

Since macrophages are a part of the innate immune system, they are crucial in the regulation of tissue homeostasis and instrumental in orchestrating inflammatory responses during disease [115]. Activated immune cells produce IL-10, an anti-inflammatory cytokine [116] that controls macrophage functions [117]. For example, IL-10 treatment converts blood monocytes into M2 macrophages [118], which are prominently involved in the clearance of early apoptotic cells [119]. A recent report specifies that IL-10 is an important anti-inflammatory cytokine that controls the metabolic switch to glycolysis that occurs in macrophages, suppresses mTORC1 activity, and eliminates dysfunctional mitochondria [120]. The immunosuppressive cytokine IL-10 regulates the production of autocrine IL-10 and nitric oxide in macrophages [121]. Together, these results strongly suggest that Akt1 controls the production of IL-10 and the increased expression of the *Il10* gene in Akt1^only^ macrophages apparently provides a survival advantage over Akt3^only^ cells.

Given that the PI3K pathway is frequently altered in human cancer [122] and increased PI3K signaling is a hallmark of cancer [5], a large number of isoform-selective and isoform-sparing PI3K inhibitors have been designed and are being tested in oncology clinical trials to suppress pathological survival of cancer cells [5,123,124,125,126]. However, this approach also suppresses PI3K/Akt activity [127,128] and the survival of non-cancer cells [129,130]. Early cardiovascular and metabolic sequelae from PI3K inhibitors include hyperglycemia and electrocardiographic disturbances in the heart [127,128]. It is possible that systemic administration of PI3K/Akt inhibitors may be associated with increased apoptosis in macrophages and, at early stages of atherosclerosis, may suppress lesion growth. In contrast, increased apoptosis induced by PI3K/Akt inhibitors in advanced lesions may contribute to the formation of increased necrotic cores and complicated atherosclerotic lesions. These considerations are particularly important because the advent of targeted cancer therapies has resulted in a growing number of cancer survivors, numbering 17,000,000 in the United States alone [128]. Cardiovascular disease—especially atherosclerosis—is a major cause of mobility and mortality in this population. Therefore, it is critically important to elucidate the molecular details and physiological relevance of macrophage PI3K/Akt signaling in atherosclerosis in this population.
